# A noninvasive nomogram model based on CT features to predict DNA mismatch repair deficiency in gastric cancer

**DOI:** 10.3389/fonc.2023.1066352

**Published:** 2023-03-09

**Authors:** Jie-Yu Chen, Ya-Han Tong, Hai-Yan Chen, Yong-Bo Yang, Xue-Ying Deng, Guo-Liang Shao

**Affiliations:** ^1^ Department of Radiology, Zhejiang Cancer Hospital, Institute of Basic Medicine and Cancer (IBMC), Chinese Academy of Sciences, Hangzhou, Zhejiang, China; ^2^ Department of Interventional Radiology, Zhejiang Cancer Hospital, Institute of Basic Medicine and Cancer (IBMC), Chinese Academy of Sciences, Hangzhou, Zhejiang, China; ^3^ Clinical Research Center of Hepatobiliary and Pancreatic Diseases of Zhejiang Province, Hangzhou, China

**Keywords:** gastric cancer, mismatch repair deficiency, microsatellite instability, tomography, nomograms

## Abstract

**Objectives:**

DNA mismatch repair deficiency (dMMR) status has served as a positive predictive biomarker for immunotherapy and long-term prognosis in gastric cancer (GC). The aim of the present study was to develop a computed tomography (CT)-based nomogram for preoperatively predicting mismatch repair (MMR) status in GC.

**Methods:**

Data from a total of 159 GC patients between January 2020 and July 2021 with dMMR GC (n=53) and MMR-proficient (pMMR) GC (n=106) confirmed by postoperative immunohistochemistry (IHC) staining were retrospectively analyzed. All patients underwent abdominal contrast-enhanced CT. Significant clinical and CT imaging features associated with dMMR GC were extracted through univariate and multivariate analyses. Receiver operating characteristic (ROC) curve analysis, decision curve analysis (DCA) and internal validation of the cohort data were performed.

**Results:**

The nomogram contained four potential predictors of dMMR GC, including gender (odds ratio [OR] 9.83, 95% confidence interval [CI] 3.78-28.20, P < 0.001), age (OR 3.32, 95% CI 1.36-8.50, P = 0.010), tumor size (OR 5.66, 95% CI 2.12-16.27, P < 0.001) and normalized tumor enhancement ratio (NTER) (OR 0.15, 95% CI 0.06-0.38, P < 0.001). Using an optimal cutoff value of 6.6 points, the nomogram provided an area under the curve (AUC) of 0.895 and an accuracy of 82.39% in predicting dMMR GC. The calibration curve demonstrated a strong consistency between the predicted risk and observed dMMR GC. The DCA justified the relatively good performance of the nomogram model.

**Conclusion:**

The CT-based nomogram holds promise as a noninvasive, concise and accurate tool to predict MMR status in GC patients, which can assist in clinical decision-making.

## Introduction

Gastric cancer (GC) was the fifth most common malignancy and the fourth leading cause of cancer deaths globally in 2020 and remains an aggressive and important cancer worldwide, especially in Asian countries (1, 2). GC carries a poor prognosis and shows marked complexity and heterogeneity in clinical characteristics and response to treatments. Recently, underlying molecular classification driving differences in treatment outcomes has been proposed by The Cancer Genome Atlas (TCGA) project and the Asian Cancer Research Group (ACRG) (3, 4). Microsatellite instability (MSI) with a high mutational load is one of the four molecular subtypes of GC (3, 4). MSI refers to the accumulation of repetitive insertion or deletion mutations in short repetitive DNA sequences as a consequence of DNA mismatch repair deficiency (dMMR), which leads to failure in repairing the errors (5).

A growing body of evidence has supported that patients with MSI-high (MSI-H)/dMMR GC have impressive and durable responses to immune checkpoint inhibition and survival benefits from that (6–10). MSI-H status is an inversely negative predictive factor for neoadjuvant/adjuvant chemotherapy in resectable GC (11–13). Therefore, MSI/mismatch repair (MMR) status has remarkable clinical utility. Universal testing for MSI by polymerase chain reaction (PCR) or MMR status using IHC in GC is recommended in all newly diagnosed patients by the National Comprehensive Cancer Network (NCCN) and the Chinese Society of Clinical Oncology (CSCO) guidelines (14, 15). The incidence of MSI-H GC varies between 8%-22% among different countries and ethnicities (16, 17). However, PCR and immunohistochemistry (IHC) have low cost-effectiveness because of the relatively low prevalence of MSI-H/dMMR and are usually performed postoperatively or require sufficient preoperative biopsy tissue.

In contrast, computed tomography (CT) scan which is readily available and noninvasive is used routinely for preoperative evaluation of GC. CT provides morphological information about primary tumors and locoregional/metastatic spread of the disease. Previous studies have shown that tumors with different MSI/MMR statuses have some differences in CT image features (18, 19), but screening for dMMR GC has ignored that. Therefore, we aimed to develop a robust and user-friendly nomogram based on noninvasive gastric CT scans for the preoperative identification of dMMR GC patients.

## Materials and methods

### Patients

**Figure 1:**
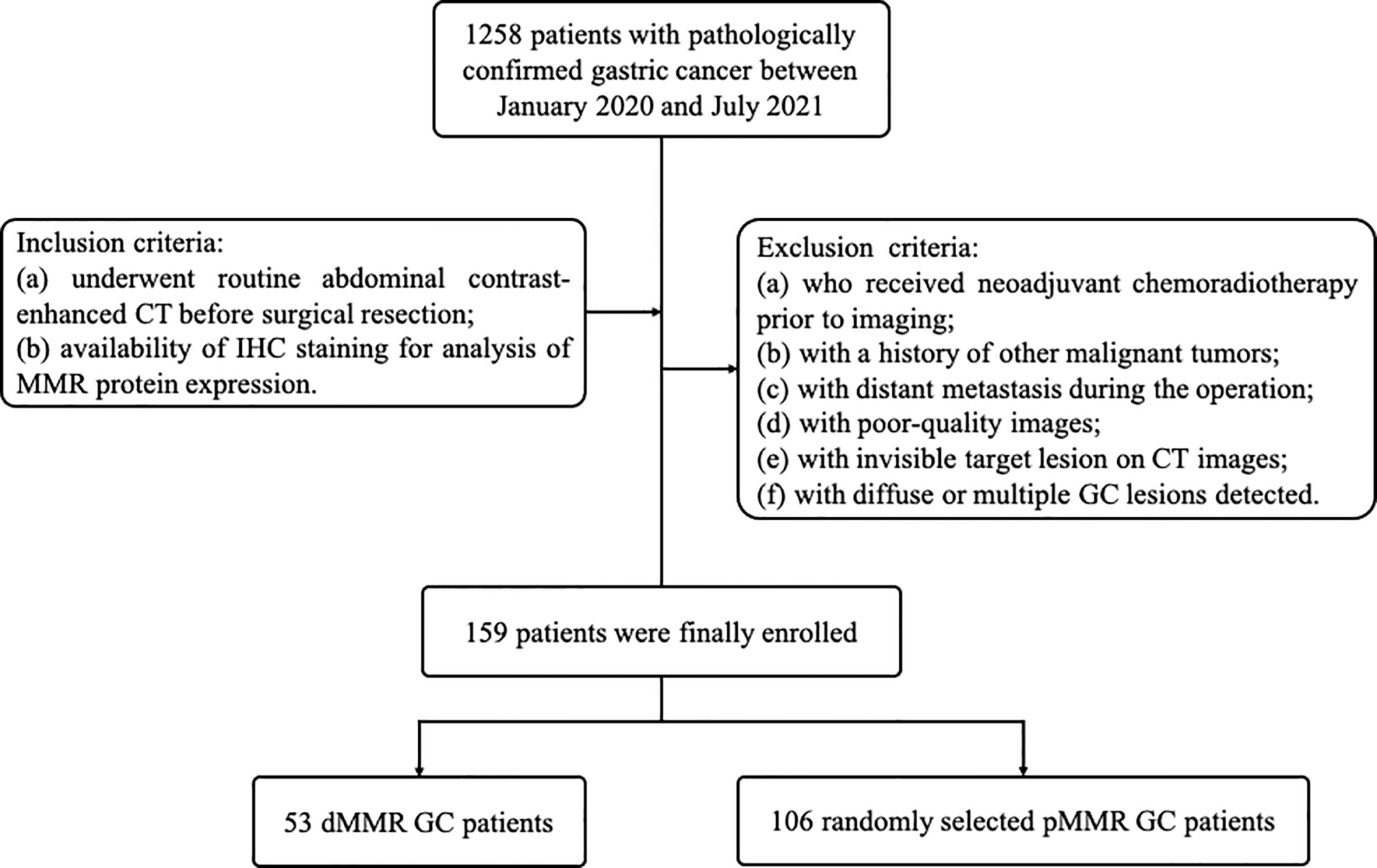
Flowchart of the patient selection and patient exclusion.

Our institutional review board approved this retrospective study, and the requirement for written informed consent was also waived. Initially, we performed a search of electronic medical records between January 2020 and July 2021 and identified a total of 1258 consecutive patients with surgically confirmed GC. Inclusion criteria were as follows: (a) underwent routine abdominal contrast-enhanced CT before surgical resection; (b) availability of IHC staining for analysis of MMR protein expression. Patients (a) who received neoadjuvant chemoradiotherapy prior to imaging; (b) with a history of other malignant tumors; (c) with distant metastasis during the operation; (d) with poor-quality images; (e) with invisible target lesions on CT images and (f) with diffuse or multiple GC lesions detected were excluded. The final study cohort comprised 53 dMMR patients and 106 MMR-proficient (pMMR) patients who were randomly selected (**Figure 1**).

### Pathological analysis

The postoperative pathological diagnosis and immunohistochemical staining were judged by two experienced pathologists. Tumor histological type, localization, ulceration, degree of differentiation, invasion depth and lymphovascular invasion were evaluated according to the 8th edition of AJCC cancer staging criteria of GC.

IHC for MMR protein (MLH1, MSH2, MSH6, and PMS2) expression was used to determine the MMR status and routinely performed by a standard streptavidin biotin-peroxidase procedure. Tumors displaying loss of at least one MMR protein were collectively referred to as dMMR, and those with intact expression were referred to as pMMR.

### CT images acquisition

Abdominal contrast-enhanced CT examinations of patients were performed using a 64-channel CT (SOMATOM Definition Flash; Siemens Healthcare, Erlangen, Germany). Patients were instructed to fast for at least 6 hours prior to CT examination. Twenty minutes before scanning, patients were intravenously administered 10 mg of anisodamine to minimize gastrointestinal peristalsis and then drank 800-1000 ml water to expand the stomach. The CT scans were performed with the patient in the supine position and covered the entire abdomen from the diaphragmatic dome to the pubic symphysis. The images were acquired with the following parameters: a tube voltage 120 of kVp, a tube current of 200 mAs, detector collimation of 0.6 mm, an image matrix of 512 × 512, a slice thickness of 5 mm, a slice interval of 5 mm, and a pitch of 0.6 mm. After the unenhanced scan, 100 ml of iodinated contrast agent was administered at a rate of 3 ml/s into the antecubital vein with an automatic injector pump. The arterial and portal venous phases were obtained at 30-35 s and 65-70 s after injection of contrast material, respectively. The enhanced axial CT images were reconstructed with a section thickness of 1 mm for multiplanar reformation (MPR) reconstruction.

### Image interpretation

All the obtained images were evaluated retrospectively by two experienced abdominal radiologists (with 10 and 6 years of experience in abdominal radiology) who were blinded to the histopathological results. All variables included clinical data, morphological features, and CT quantitative parameters. Morphological feature analysis was independently performed by two radiologists, and the final results were determined by consensus, while the acquisition of quantitative parameters was jointly completed by both radiologists. The variables on CT were defined and measured as follows: tumor location (upper third of the stomach, middle third of the stomach and lower third of the stomach), tumor size and tumor thickness (the longest and thickest diameter of tumor on axial, sagittal, or coronal CT image), surface ulceration (absent or present), adjacent organ invasion (absent or present), growth pattern (localized or infiltrative type), and the largest lymph node size (short-axial diameters of the largest lymph node < 0.8 cm or ≥ 0.8 cm). A circular region of interest (ROI, a least 20 mm2 for large lesions and a circular with diameter slightly smaller than tumor thickness for small ones) was positioned to encompass as much of the most strongly enhanced portion of the tumor and the abdominal aorta as possible at the same slice of the portal venous phase by the two radiologists together. The average CT attenuation values of each tumor parenchyma (CTAVtumor) and abdominal aorta (CTAVaorto) were finally obtained to calculate the normalized tumor enhancement ratio (NTER) using the following formula: NTER (%) = CTAVtumor/CTAVaorto * 100. Additionally, other clinical data were recorded, including age, gender, hypertension and so on.

### Statistical analysis

Continuous data distributions were verified using the Shapiro−Wilk test. Normally distributed data were presented as the mean ± standard deviation, and data with a nonnormal distribution were presented as medians and ranges (25th, 75th percentiles). Categorical variables were expressed as frequencies and percentages. In univariate analysis, differences between the two groups were compared using Student’s t test or the Mann−Whitney U test for continuous data and using the chi-square or Fisher’s exact test for categorical variables. Receiver operating characteristic (ROC) curve analysis was employed to evaluate the predictive value of each continuous parameter, which showed statistically significant differences and aided in determining their optimal cutoff value. Subsequently, the significant variables from the univariate analysis were entered into the multivariate binary logistic regression using the stepwise method. Finally, a nomogram was generated based on the identified independent predictors. The nomogram was internally validated by bootstrapping with 1000 resamples. To quantify the model performance in predicting MMR status, we assessed model discrimination using the AUC (area under the curve) and calibration using a calibration curve combined with the Hosmer−Lemeshow test. Additionally, the decision curve analysis (DCA) was utilized to determine the clinical practicability of the nomogram.

All data were processed in SPSS software (version 26.0.0; IBM Corp., Armonk, NY, USA) and R software (version 4.1.2; R Foundation for Statistical Computing, Vienna, Austria). A two-sided p value of < 0.05 indicated statistical significance.

## Results

### Clinical characteristics of the study patients

Of the 1258 consecutive GCs, 97 (7.7%) patients had dMMR status. A total of 159 patients, comprising 53 with dMMR and 106 with pMMR, were enrolled in this study. The demographic and clinical characteristics of the patients are summarized in **Table 1**. GCs with dMMR were more likely to be older, female and have a N0 stage. There were no significant differences with regard to hypertension, histological differentiation degree or T stage between groups.

**Table 1:** Demographic and clinical characteristics of the study patients.

	pMMR (n=106)	dMMR (n=53)	P value
Age (yr)	64 (57.0, 69.0)	70 (64.0, 75.5)	<0.001^*^
Gender (%)			< 0.001
Male	91 (85.85)	22 (41.51)	
Female	15 (14.15)	31 (58.49)	
**Hypertension (%)**			0.816
No	68 (64.15)	33 (62.26)	
Yes	38 (35.85)	20 (37.74)	
Histological differentiation degree (%)			0.059^†^
Adenocarcinoma			
Poorly differentiated	75 (70.75)	45 (84.91)	
Well-/moderately differentiated	22 (20.75)	5 (9.43)	
Mucinous carcinoma	2 (1.89)	1 (1.89)	
Signet ring cell carcinoma	6 (5.66)	0 (0)	
Adenosquamous carcinoma	1 (0.94)	2 (3.77)	
T stage (%)			0.293
T1-2	35 (33.02)	22 (41.51)	
T3-4	71 (66.98)	31 (58.49)	
N stage (%)			0.002
N0	30 (28.30)	28 (52.83)	
N1+	76 (71.70)	25 (47.17)	

*Mann-Whitney U-test; † Fisher’s exact test.

### Comparison of CT features between dMMR and pMMR GC patients

Differences in CT features between dMMR and pMMR GC patients are presented in **Table 2**. Significant differences were observed in location, surface ulceration, adjacent organ invasion, tumor size and NTER. The tumor size of the dMMR tumors was substantially larger, and the NTER was lower than that of pMMR tumors. Well-defined margins, thicker lesions and short-axial diameters of the largest lymph node ≥ 0.8 cm were found more often in dMMR patients, but the difference was not statistically significant (P = 0.094, P= 0.084, and P = 0.050, respectively).

**Table 2:** Comparison of CT features between dMMR and pMMR GC patients.

	pMMR (n=106)	dMMR (n=53)	P value
Location (%)			0.001
Upper	34 (32.08)	3 (5.66)	
Middle	12 (11.32)	7 (13.21)	
Lower	60 (56.60)	43 (81.13)	
Margin (%)			0.094
Well-defined	30 (28.30)	22 (41.50)	
Ill-defined	76 (71.70)	31 (58.50)	
Surface ulceration (%)			0.009
No	11 (10.38)	14 (26.42)	
Yes	95 (89.62)	39 (73.58)	
Adjacent organ invasion (%)			0.025
No	75 (70.75)	46 (86.79)	
Yes	31 (29.25)	7 (13.21)	
Tumor size (cm)	3.8 (3.0, 5.2)	5.4 (3.2, 7.0)	0.008*
Tumor thickness (cm)	1.2 (0.8, 1.6)	1.40 (0.9,1.7)	0.083*
NTER (%)	72.92 ± 10.18	62.58 ± 10.65	<0.001
The largest lymph node size (%)			0.050
< 0.8 cm	71 (66.98)	27 (50.94)	
≥ 0.8 cm	35 (33.02)	26 (49.06)	

*Mann-Whitney U-test.

### Analyzing dMMR risk factors and building a CT-based predictive nomogram

*Post-hoc* comparison revealed that dMMR tumors were significantly more frequently found in the upper stomach than in the middle and lower regions, while there was no significant differences between tumors in the middle and lower stomach. Thus, the latter two subgroups were pooled into a single group for subsequent comparisons, hereafter named the “middle-lower” group. Then, we organized the continuous variables into meaningful and dichotomous categories according to the optimal cutoff value by ROC curve (**Table 3**).

**Table 3:** ROC curve analysis results for classification of the patients.

Parameters	Cutoff value	AUC	95%CI	Sensitivity	Specificity	P value
Age (yr)	66.5	0.700	0.614-0.787	71.7%	67.9%	< 0.001
Tumor size (cm)	5.55	0.629	0.529-0.729	47.2%	83.0%	0.008
NTER (%)	67.49	0.772	0.692-0.851	71.7%	77.4%	< 0.001

AUC, area under the curve; CI, confidence interval.

**Figure 2:**
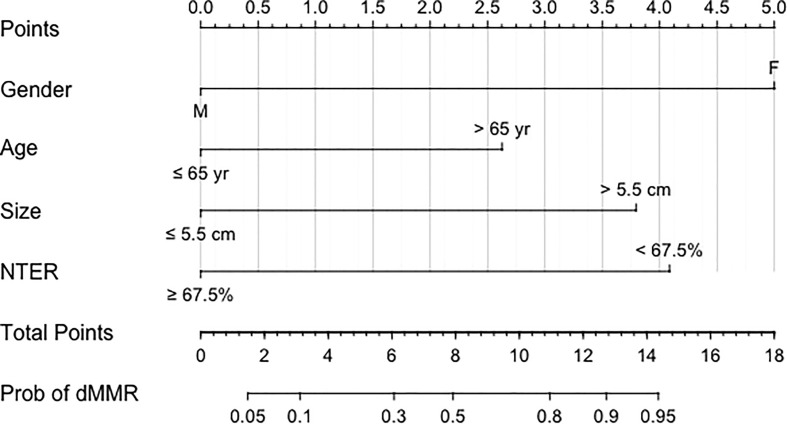
CT-based preoperative nomogram for dMMR risk prediction in GC patients.

To select final predictors, eight candidate predictors with a P ≤ 0.05 in the univariate logistic analysis were included when performing the stepwise logistic regression analysis. Gender, age, tumor size and NTER emerged as significant independent predictors of the dMMR phenotype in GC patients (**Table 4**). Based on the multivariate analysis results, a predictive nomogram incorporating the above four factors was then constructed (**Figure 2**).

**Table 4:** Risk factors for predicting dMMR tumor in GC.

Subgroups	pMMR (%)	dMMR (%)	Univariate analysis P value		Multivariate analysis OR (95%CI)	P value
Gender			< 0.001	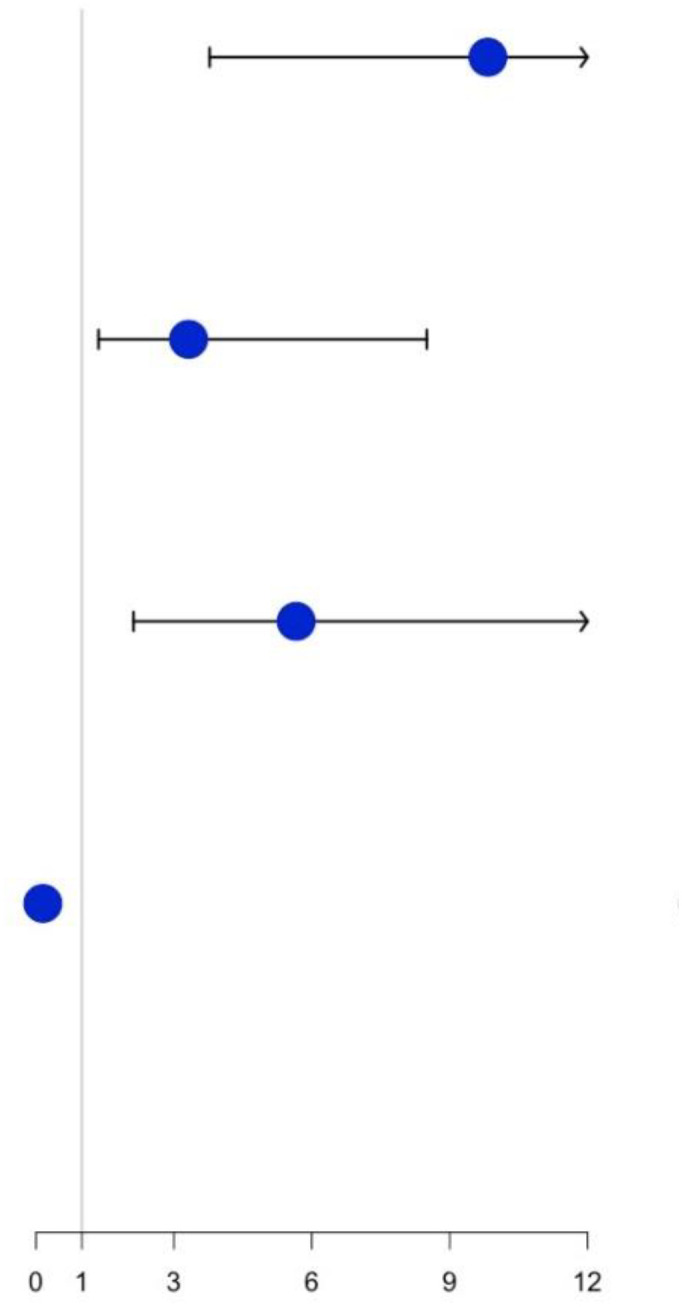		< 0.001
Male	91 (85.85)	22 (41.51)			Reference	
Female	15 (14.15)	31 (58.49)			9.83 (3.78, 28.20)	
Age			< 0.001			0.010
≤ 65 yr	69 (65.09)	15 (28.30)			Reference	
> 65 yr	37 (3491)	38 (71.71)			3.32 (1.36, 8.50)	
Tumor size			< 0.001			< 0.001
≤ 5.5 cm	88 (83.02)	28 (52.83)			Reference	
> 5.5 cm	18 (16.98)	25 (47.17)			5.66 (2.12, 16.27)	
NTER			< 0.001			< 0.001
< 67.50%	76 (71.70)	12 (22.64)			Reference	
≥ 67.50%	30 (28.30)	41 (77.36)			0.15 (0.06, 0.38)	
Location			0.001			/
Upper	34 (32.08)	3 (5.66)			Reference	
Middle-lower	72 (67.92)	50 (94.34)			/	

OR, odds ratio; CI, confidence interval.

### Performance and validation of the nomogram

**Figure 3:**
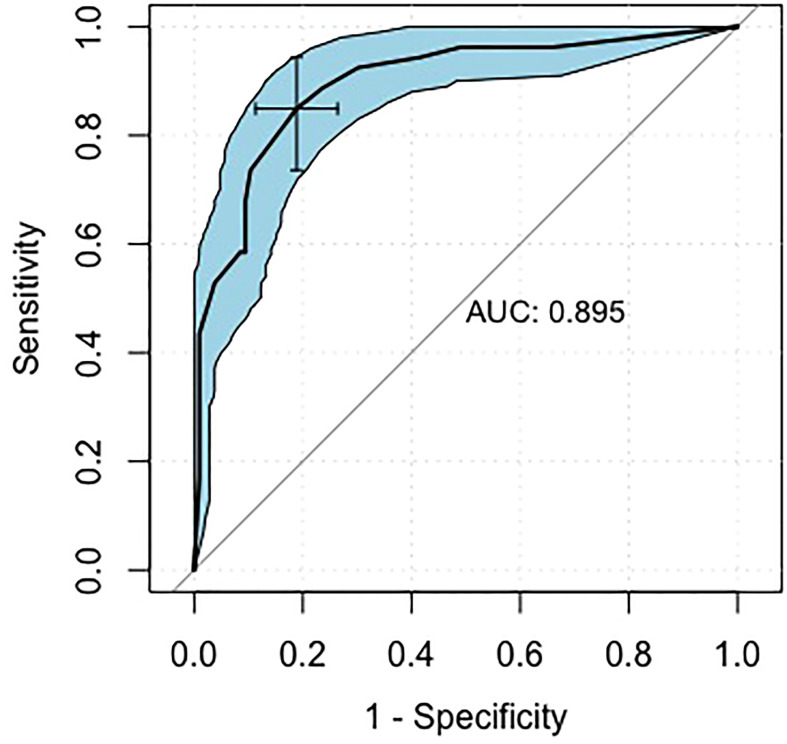
Receiver operating characteristic curves of the nomogram. AUC: area under the curve.

**Figure 4:**
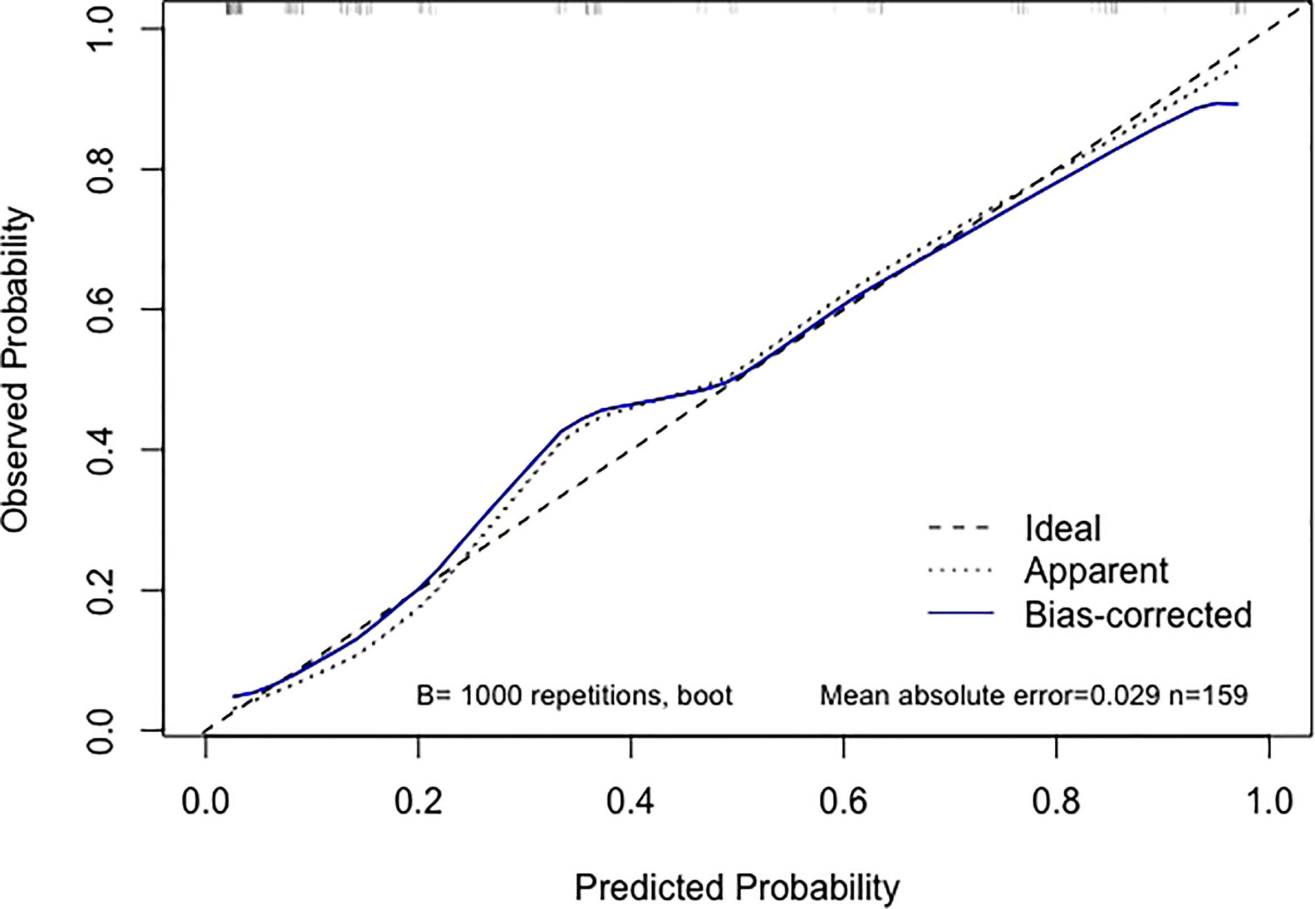
Calibration curve of the nomogram. When the solid line (representing the nomogram) was closer to the the dotted line (“Ideal”, representing an ideal model), the predictive performance of the nomogram was better.

**Figure 5:**
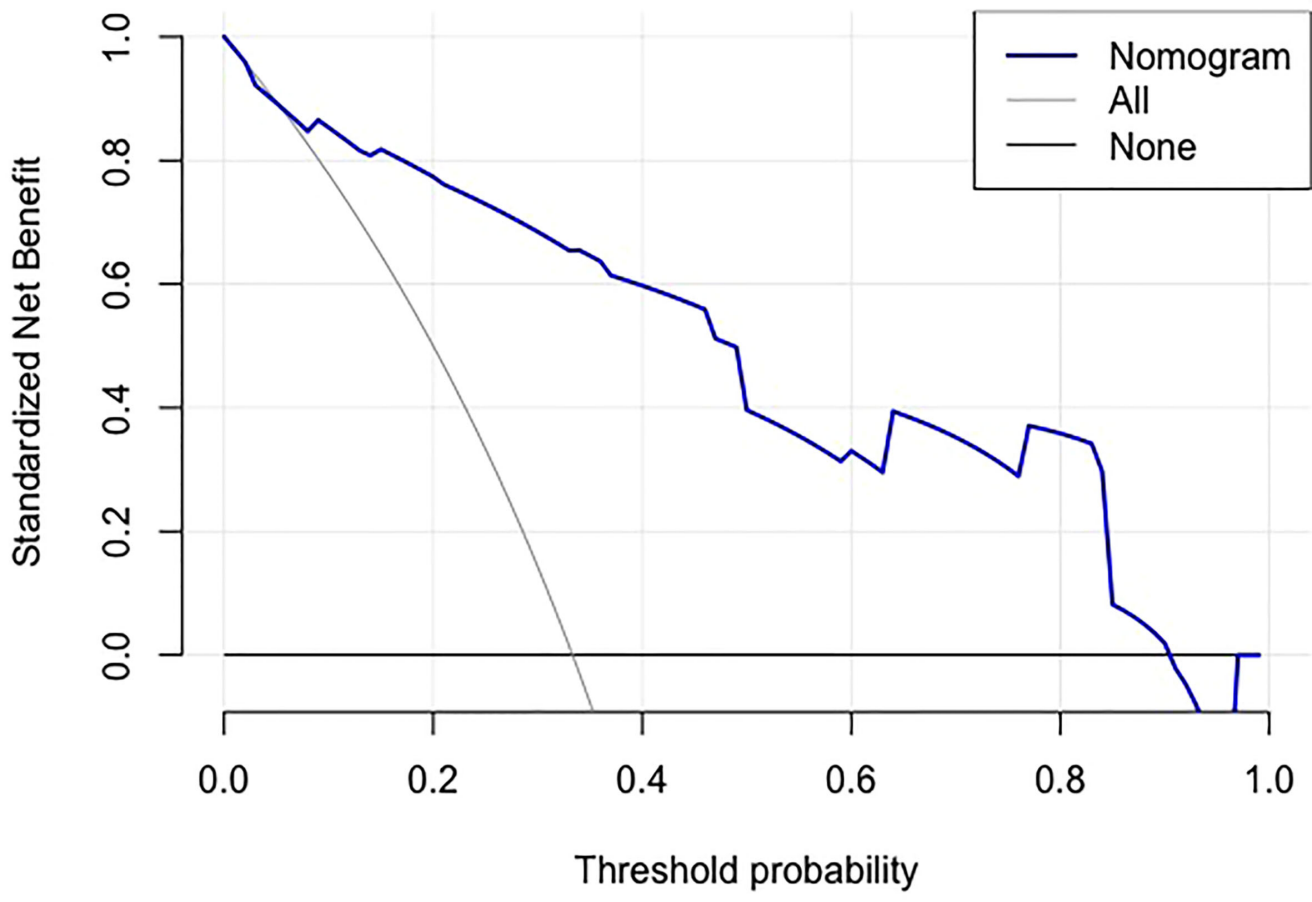
Decision curve analysis of the nomogram.The gray line is for all patients with dMMR, the black line line is for no patients were dMMR, and the blue line represents the nomogram model. The graph depicts the expected net benefit per patient relative to the nomogram prediction of dMMR risk. The farther the blue line is to the grey and black lines, the better clinical value the nomogram holds.

Using an optimal cutoff value of 6.6 points, the nomogram provided better AUC values than each risk factor for dMMR (AUC = 0.895) (**Figure 3**), with a sensitivity, specificity, NPV, PPV and accuracy of 84.91%, 81.13%, 91.49%, 69.23% and 82.39%, respectively. To further evaluate the predictive ability of this model without overfitting, the corrected AUC obtained by internal validation using the bootstrap method was 0.896 (95% CI 0.837-0.950). The calibration curve demonstrated good consistency between the predicted risk and observed dMMR probability (**Figure 4**), and the Hosmer−Lemeshow test also indicated that there was no departure from a perfect fit (P = 0.945). Furthermore, the DCA justified relatively good performance for the nomogram model according to clinical application (**Figure 5**).

## Discussion

The clinical significance of MSI/MMR status in GC patients, which can serve as a positive predictive biomarker for immunotherapy and long-term prognosis (11), has become widely recognized worldwide. Many consequent attempts at developing prediction models based on pathological slices or findings to predict MSI-H/dMMR in GC have been made in recent years. Kather et al. (2), Muti et al. (20) and Valieris et al. (21) proposed deep learning classifiers for detecting MSI/MMR status in GC from digital histological images and confirmed their good performance. Additionally, a prediction model presented in the study conducted by Suzuki et al. (22) involved pathological T and M stage. Immune checkpoint inhibitors are recommended by the guidelines for unresectable locally advanced or metastatic GCs with MSI-H/dMMR (14). The patients are not candidates for surgical treatment and could not obtain definitive pathological diagnosis. Based on this, we established a robust nomogram model based on radiologic features to preoperatively and noninvasively identify dMMR GCs.

Our nomogram with favorable discrimination (AUC = 0.895) and calibration was internally confirmed to be relatively stable through bootstrap validation. A multivariate set of predictors identified by logistic regression analysis comprised gender, age, tumor size, and NTER, of which the latter two could be available as a result of routine enhanced CT. GC with dMMR was more prevalent in female and older patients in the current cohort, which was almost well recognized by previous studies (11, 16, 22–25). Recent reliable evidence suggests that the most common age cutoff value is 65 or 70 years (16, 22), while ours is 65 years.

Other known strong predictors included tumor size and NTER measured by enhanced CT. Given that the stomach is a hollow organ, the gastric filling state increases the uncertainty of the evaluation of tumor size. The contribution of tumor size to the prediction of MMR status in GC remains controversial. Seo et al. (16) reported that GC with MSI-H was more likely associated with a larger tumor size (≥ 5 cm), while some research suggested that there was no significant difference in tumor size between the two groups (25, 26). Additionally, a research group evaluated tumor size through tumor thickness by CT and revealed that the CT tumor thickness of the dMMR group was less than that of the pMMR group (18). However, a nonsignificant difference was seen between the two groups with respect to tumor thickness in this study. This study reported a cutoff value close to that reported in previous studies on tumor size in GC. The enhancement patterns of lesions are very important CT features. Our study demonstrated that the NTER of dMMR GC was significantly lower than that of pMMR GC. In other words, dMMR GC presents a lower degree of enhancement and less blood supply than pMMR GC. Wu et al. (19) obtained analogous results in colorectal cancer (CRC) in which the MSI CRC had significantly lower normalized iodine concentration values in dual-energy CT. This discrepancy may be partially explained by differences in microscopic features. In the 2000s and 2010s, it was demonstrated that vascular endothelial growth factor expression and microvessel count were lower in MSI gastric and colon carcinomas than in their microsatellite stable (MSS) counterparts (27–29). Also, in line with previous research (16, 22, 25), tumor location differed significantly between the two groups. Nevertheless, it was not an independent predictor for dMMR GC in this study. A study of Fan et al. (30) suggested that hypertension is one of the clinical features with high predictive value to discriminate low- and high-MSI expression of CRC and occurs with greater frequency in MSI-H CRC. However, similar results were not observed in this study and another Chinese study (31).

Using an optimal cutoff value of 6.6 points, the user-friendly and well-fitted nomogram achieved a sensitivity of 84.91% and a specificity of 81.13% to detect the presence of dMMR in our study populations. For convenience, we could transform that into an integral model to make it more applicable to clinical work: 5 points for female patients; 3 points for older patients; 4 points for larger tumors; and 4 points for tumors having lower NTER. Total points of ≤ 4, 5-11, and ≥ 12 corresponded to probabilities of dMMR of 5.13%, 59.10%, and 96.97%, respectively. Likewise, the high NPV (91.49%) of the nomogram may contribute to the recognition of patients at higher risk for non-dMMR events, which may reduce the time and cost involved in identifying dMMR GC.

This study has several limitations. First, its selection bias and other inherent weaknesses associated with a retrospective observational study were difficult to avoid. Second, few studies have observed that multiple GCs have a relatively high incidence of MSI-H/dMMR than solitary GCs (16, 32). But given the extremely low number of multiple GC cases, we excluded those patients. This may have had an impact on the outcomes. Third, the study was conducted in only one single tertiary medical center, and the sample size was not sufficiently large. As such, future studies must focus on involving a larger cohort and external validation at multiple centers to fully substantiate the transportability and generalizability of the presented nomogram.

In conclusion, our proposed nomogram model incorporating clinical and CT features shows satisfactory performance and makes preoperative prediction of dMMR in GC possible. This might provide clinicians with an easily available and cost-effective tool to assist in clinical decision-making, such as obtaining adequate endoscopic samples for detection and even immunotherapy. In the future, multicenter large-scale validation of the screening model justifies further study.

## Data availability statement

The original contributions presented in the study are included in the article/supplementary material. Further inquiries can be directed to the corresponding authors.

## Author contributions

J-YC designed the study, completed the statistics and wrote original manuscript. Y-HT and H-YC participated in its design and collected the clinical and imaging data. Y-BY collect the cases and helped in the data presentation and result interpretation. X-YD and G-LS revised the manuscript and guaranteed the entire study. All authors contributed to the article and approved the submitted version.
